# Severe Pediatric MOGAD‐Associated ADEM With Persistent Neurological Morbidity and Later‐Onset Epilepsy of Uncertain Etiology: A Case Report

**DOI:** 10.1002/ccr3.73579

**Published:** 2026-09-23

**Authors:** Desiree‐Anne Ramsaran, Garrett Gianneschi, Janet Elgallab

**Affiliations:** ^1^ Department of Pediatrics The Brooklyn Hospital Center Brooklyn New York USA; ^2^ Department of Neurology Rutgers‐New Jersey Medical School Newark New Jersey USA

**Keywords:** ADEM, case report, epilepsy, MOGAD, MOG‐IgG, pediatric neurology

## Abstract

This report describes a 7‐year‐old boy with MOGAD presenting as severe ADEM with fever, encephalopathy, seizures, markedly elevated intracranial pressure, diffuse multifocal MRI abnormalities, and serum MOG‐IgG positivity. He required intravenous corticosteroids, plasma exchange, IVIG, and tocilizumab, followed by rehabilitation and maintenance IVIG. Persistent sequelae included dysarthria, memory and learning difficulties, and ADHD diagnosed after the acute illness. Approximately 3 years later, he developed frequent hyperkinetic seizures with bifrontal‐predominant multifocal epileptiform abnormalities and subsequently achieved seizure freedom on combination therapy. Follow‐up contrast‐enhanced MRI after epilepsy onset demonstrated multifocal encephalomalacia/gliosis and diffuse cerebral volume loss, without acute infarct, hemorrhage, mass effect, hydrocephalus, abnormal enhancement, or hippocampal abnormality. Prior inflammatory brain injury is therefore a plausible acquired substrate, but an independent genetic, traumatic, or other epilepsy predisposition cannot be excluded. The case emphasizes long‐term neurodevelopmental follow‐up after severe pediatric MOGAD‐ADEM and careful etiologic evaluation when remote epilepsy emerges.

## Key Clinical Message

Severe pediatric MOGAD‐associated ADEM may leave persistent neurocognitive morbidity despite aggressive immunotherapy. When epilepsy emerges years later, prior inflammatory injury is one possible substrate but should not be assumed causal; contemporaneous neuroimaging and epilepsy‐focused genetic evaluation are important to assess alternative structural and genetic etiologies.

## 1 Introduction

Myelin oligodendrocyte glycoprotein antibody‐associated disease (MOGAD) is an autoimmune demyelinating disorder of the central nervous system that frequently affects children [1]. It commonly presents with acute disseminated encephalomyelitis (ADEM), optic neuritis, or transverse myelitis [2]. ADEM is a severe inflammatory disorder of the central nervous system that often follows infection and, less commonly, vaccination [1].

The prognosis of pediatric MOGAD is variable, ranging from near‐complete recovery to persistent cognitive, behavioral, speech, and motor morbidity; epilepsy has also been described in a subset of children after ADEM [2, 3, 4]. Standardized treatment algorithms remain incompletely defined, and the role of serial MOG‐IgG testing in long‐term management and relapse prediction remains debated [5]. This report describes a severe pediatric MOGAD‐associated ADEM course requiring multimodal immunotherapy and prolonged recovery, followed years later by epilepsy. The central clinical lesson is not that MOGAD caused the later epilepsy, but that remote epilepsy after a severe acquired inflammatory brain injury requires longitudinal follow‐up and a renewed etiologic evaluation that considers structural, genetic, and other competing explanations.

## 2 Case History/Examination

On Day 1, a 7‐year‐old Hispanic male with no prior medical history presented to the emergency department with headache, photophobia, hyperacusis, one episode of non‐bloody, non‐bilious vomiting, myalgia, and chills. He had a temperature of 100.7°F and a white blood cell count of 19.1 k/mm^3^, was diagnosed with a viral illness, and was discharged with NSAIDs.

Three days later, he returned minimally responsive with similar symptoms and progressively worsening mental status. Vital signs were labile over the subsequent 24 h, with blood pressure ranging from normotensive values to 170/116 mmHg, heart rate 62–167 beats/min, respiratory rate 18–39 breaths/min, and a maximum temperature of 103.2°F. On examination, he was stuporous, opened his eyes spontaneously, withdrew to pain, vocalized incomprehensible sounds, and had a generalized tonic–clonic seizure lasting approximately 30 s, prompting intubation for airway protection. He was intermittently agitated and appeared uncomfortable, with episodic decerebrate posturing and a Glasgow Coma Scale score of 10.

## 3 Differential Diagnosis, Investigations, and Treatment

**FIGURE 1:**
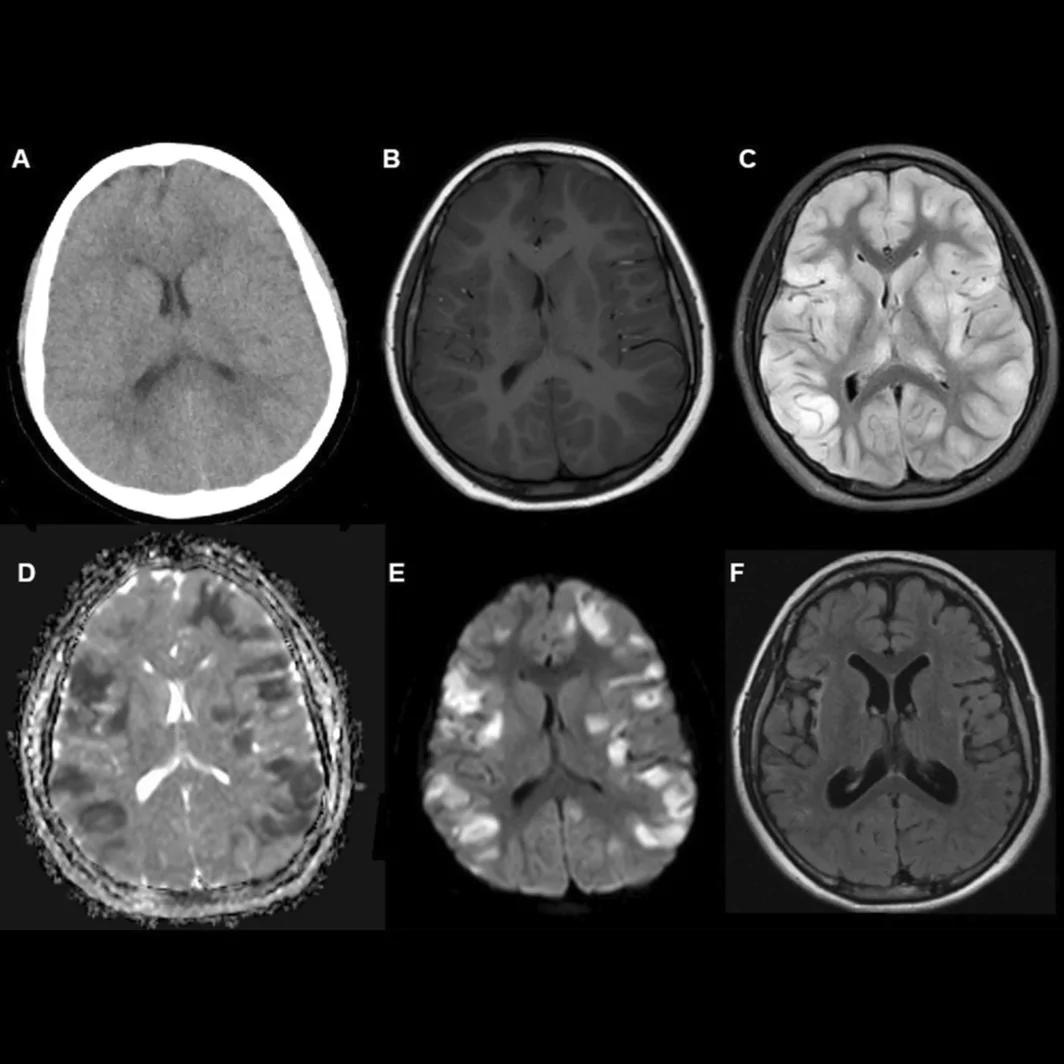
(A) Normal head CT at initial presentation. (B–E) Brain MRI with and without contrast, including T1, T2/FLAIR, ADC, DWI, and post‐contrast sequences, obtained 5 days after symptom onset, demonstrating scattered bilateral diffusion restriction involving the frontal, parieto‐occipital, and temporal lobes, with cytotoxic edema/gyral swelling and additional deep gray/right pontine signal abnormality; no abnormal enhancement was seen. (F) T2/FLAIR MRI 16 months after initial presentation demonstrating hyperintensities involving the bilateral insula, superior frontal lobes, bilateral temporal lobes, and left posterior parietal lobe, with signal abnormality in the left putamen and adjacent cortical atrophy.

Laboratory studies were notable for leukocytosis (27.8 k/mm^3^) with neutrophil predominance, respiratory acidosis, and a C‐reactive protein level of 5.53 mg/L. The initial head CT was normal (Figure 1A). Lumbar puncture demonstrated an unmeasurable opening pressure greater than 39 mmHg. Cerebrospinal fluid was clear and colorless, with white blood cell count 68 cells/mm^3^, red blood cell count 1 cell/mm^3^, glucose 93 mg/dL, and protein 43.3 mg/dL. The CSF differential showed 8% neutrophils, 66% lymphocytes, and 26% monocytes, with 0% eosinophils and 0% basophils. Oligoclonal band results were not available in the reviewed records and were therefore treated as not performed for this report. CSF Gram stain showed many white blood cells but no organisms, and bacterial culture showed no growth at 5 days. Fungal culture, cryptococcal testing, and acid‐fast bacilli/mycobacterial studies did not identify an organism. A CSF meningitis/encephalitis PCR panel was negative for *Escherichia coli* K1, *Haemophilus influenzae* , *Listeria monocytogenes* , *Neisseria meningitidis* , *Streptococcus agalactiae* , *Streptococcus pneumoniae* , cytomegalovirus, enterovirus, herpes simplex virus 1, herpes simplex virus 2, human herpesvirus 6, human parechovirus, varicella‐zoster virus, and *Cryptococcus neoformans* /gattii. Additional HSV DNA PCR, enterovirus RNA, and human parechovirus RNA testing were negative. SARS‐CoV‐2 testing was negative, and EBV serologies were consistent with prior exposure rather than acute EBV infection. Brain MRI with and without contrast on 4/20/2022 (Figure 1B–E) showed scattered bilateral regions of diffusion restriction involving the frontal, parieto‐occipital, and temporal lobes, with cytotoxic edema and gyral swelling, additional T2/FLAIR signal abnormality in the bilateral thalami, bilateral putamen, caudate heads, and right hemi‐pons, and no abnormal enhancement, hemorrhage, midline shift, transtentorial herniation, or hydrocephalus. Follow‐up MR angiography was noncontributory. Video EEG demonstrated diffuse delta slowing without electrographic seizures.

The initial differential diagnosis included infectious meningoencephalitis, autoimmune or inflammatory encephalitis/demyelination, toxic‐metabolic encephalopathy, and vascular or other structural causes. He was treated empirically with dexamethasone, mannitol, 3% hypertonic saline, vancomycin, ceftriaxone, acyclovir, midazolam/fentanyl, hyperventilation, and head‐of‐bed elevation to 30°. The expanded infectious evaluation did not identify a bacterial, fungal, mycobacterial, or tested viral pathogen, allowing discontinuation of antimicrobial therapy and dexamethasone. However, MOG‐IgG positivity was interpreted in the clinical and radiologic context and was not considered to exclude a preceding or concurrent infection as a potential trigger or mimic.

His examination remained largely unchanged until Hospital Day 8, when he developed asymmetric pupils and decorticate posturing. Repeat head CT demonstrated uncal herniation with cerebral edema and lateral ventricular effacement. Serum MOG‐IgG was positive at a titer of 1:100 by cell‐based immunofluorescence assay through Mayo Clinic. He was treated with intravenous methylprednisolone for 8 days followed by a taper, six sessions of plasma exchange, IVIG (2 g/kg over 2 days), and two doses of tocilizumab (8 mg/kg/dose). He regained consciousness after approximately 1 month and was discharged to inpatient rehabilitation. Table 1 summarizes selected diagnostic evaluation and findings in this case.

**TABLE 1:** Selected diagnostic evaluation in suspected pediatric MOGAD‐ADEM and findings in this case.

Domain	Evaluation	Findings in this case
Initial neuroimaging	Non‐contrast head CT to assess for hemorrhage, mass lesion, hydrocephalus, or other acute structural process; repeat imaging with neurologic deterioration or concern for raised intracranial pressure	Initial CT normal; subsequent CT demonstrated uncal herniation, diffuse cerebral edema, and ventricular effacement; acute MRI showed no hydrocephalus
CSF profile	Opening pressure; cell count with differential; protein and glucose; assessment for an inflammatory pattern	Opening pressure > 39 mmHg (unmeasurable); clear/colorless CSF; WBC 68 cells/mm^3^ with 66% lymphocytes; RBC 1 cell/mm^3^; glucose 93 mg/dL; protein 43.3 mg/dL; oligoclonal bands unavailable/not performed
Infectious studies	CSF bacterial culture; meningitis/encephalitis PCR testing; HSV testing; additional viral studies and toxicology as clinically indicated	CSF Gram stain with many WBCs but no organisms; bacterial culture no growth at 5 days; fungal, cryptococcal, and acid‐fast bacilli/mycobacterial studies negative; meningitis/encephalitis PCR panel negative for HSV‐1/2, VZV, enterovirus, human parechovirus, HHV‐6, CMV, *Cryptococcus neoformans* /gattii, and tested bacterial pathogens
MRI brain	Brain MRI with diffusion‐weighted, FLAIR, susceptibility, and contrast‐enhanced sequences when available to characterize inflammatory/demyelinating lesions, enhancement pattern, and competing structural diagnoses	Acute MRI with and without contrast showed multifocal bilateral cortical/subcortical diffusion restriction and T2/FLAIR abnormalities involving frontal, parieto‐occipital, temporal, deep gray, and right pontine regions, without abnormal enhancement. Later follow‐up imaging showed evolution to encephalomalacia/gliosis and diffuse cerebral volume loss
Vascular imaging	MRA/MRV when vasculopathy, venous thrombosis, or stroke mimic is a concern	MR angiography noncontributory
EEG	Video EEG to assess encephalopathy and exclude nonconvulsive seizures	Diffuse delta slowing; no electrographic seizures during the acute evaluation
MOG‐IgG assay	Serum cell‐based assay, interpreted with the clinical and radiologic phenotype; report quantitative titer when available	Positive MOG‐IgG by cell‐based immunofluorescence assay; titer 1:100

Follow‐up contrast‐enhanced MRIs in April and June 2022 showed interval evolution of the multifocal cortical and subcortical lesions without new lesions. The lesions decreased in size and mass effect over time, with later development of encephalomalacia, diffuse gyral/cerebral volume loss, and ex vacuo dilatation of the supratentorial ventricles. No leptomeningeal enhancement was reported, and the orbits were unremarkable.

## 4 Conclusions and Results (Outcome and Follow‐Up)

Given the severity of the acute course, he received monthly IVIG after discharge until a qualitative MOG‐IgG result through Labcorp was reported as negative 15 months after the initial presentation; no endpoint titer was provided. For the first 14 months, he received treatment at a tertiary care center, and MOG‐IgG results from that interval were not available. He also underwent neurocognitive assessment at that center, but the formal results were unavailable after transfer of care. According to the available history, he had been neurocognitively intact before ADEM. Documented residual deficits included dysarthria, memory deficits, and learning disability; ADHD was diagnosed after the acute illness, although attribution to ADEM cannot be established from the available records.

Repeat MRI 16 months after presentation showed scattered T2/FLAIR hyperintensities involving the bilateral insula, superior frontal lobes, bilateral temporal lobes, and left posterior parietal lobe, with signal abnormality in the left putamen and adjacent cortical atrophy (Figure 1F).

Approximately 3 years after the initial presentation, he developed multiple daily events concerning for seizures with frontal‐predominant features after being punched in the head by another student at school. There was no loss of consciousness or altered mentation immediately after the assault. Later that night, during dinner, he fell to the floor with full‐body choreiform movements, retained awareness, and post‐event fatigue. He also described an aura of a crawling sensation in his legs. Over the next 2 weeks, events occurred daily, lasted 10–30 s, and evolved into generalized hyperkinetic or ballistic movements and rhythmic bicycling movements with retained awareness.

**FIGURE 2:**
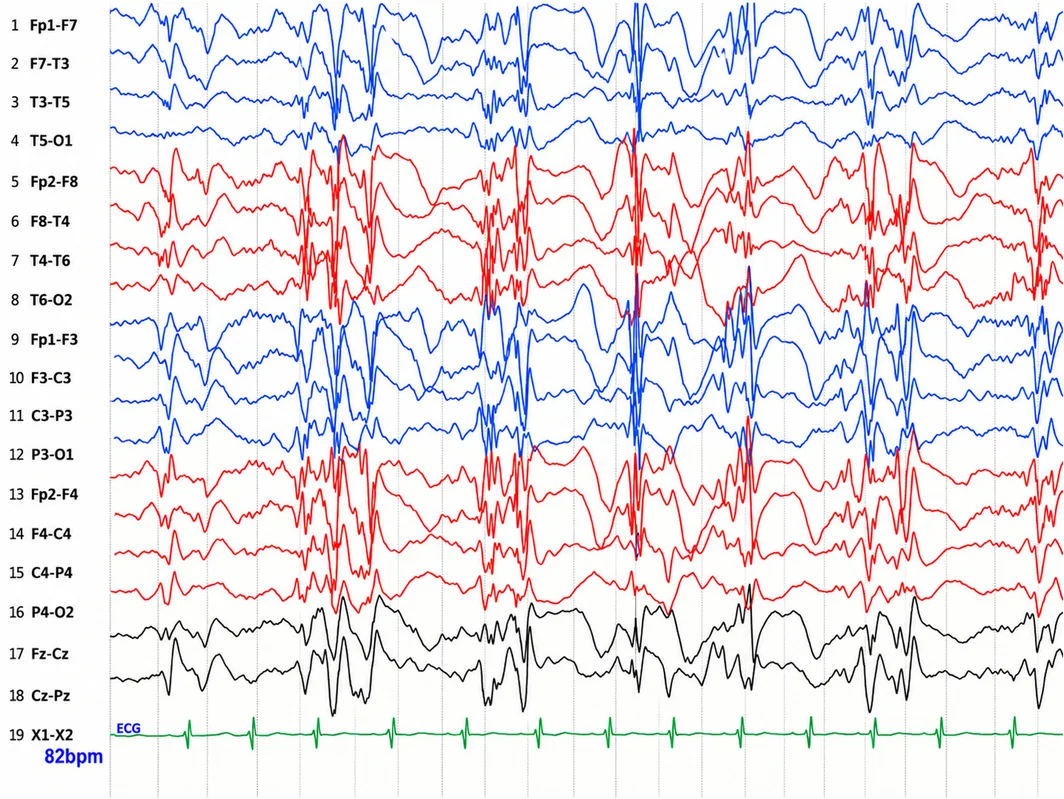
Interictal EEG recording approximately 3 years after initial presentation, demonstrating frequent bifrontal‐predominant, multifocal 2–2.5 Hz sharp‐and‐spike–wave bursts lasting more than 10 s.

**FIGURE 3:**
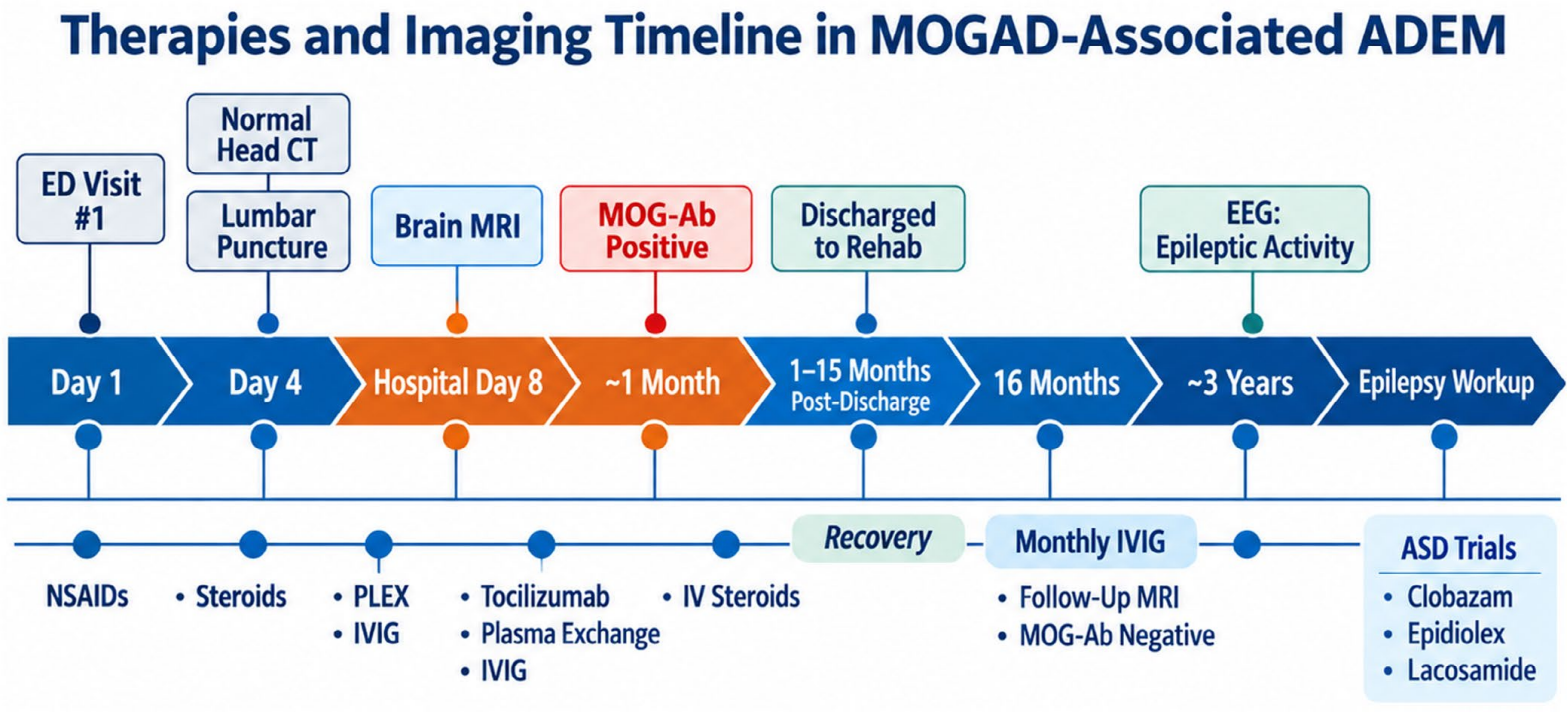
Simplified treatment and clinical timeline.

MOG‐IgG by cell‐based immunofluorescence assay through Labcorp remained negative. EEG captured seizures with diffuse electrodecrement followed by diffuse 3–4 Hz delta slowing admixed with movement artifact. Interictal EEG showed frequent bifrontal‐predominant, multifocal 2–2.5 Hz sharp‐and‐spike–wave bursts lasting 2–20 s, without clinical change, occurring more frequently in sleep and comprising approximately 25% of total sleep time (Figure 2). No immediate post‐traumatic MRI was obtained after the school assault. A follow‐up MRI brain with and without contrast obtained in August 2025 after epilepsy onset, with additional paracoronal sequences through the hippocampi, demonstrated multifocal encephalomalacia/gliosis involving the cortices and subcortical white matter of the bilateral frontal lobes, left parietal lobe, right greater than left lateral temporal lobes, bilateral insula, and left basal ganglia, with diffuse cerebral parenchymal volume loss. The hippocampi were symmetric and normal in signal intensity with preserved architecture. There was no acute infarct, acute intracranial hemorrhage, significant mass effect, midline shift, hydrocephalus, intracranial mass lesion, or abnormal intracranial enhancement. A thin gliotic tract with hemosiderin staining was present in the high right frontal parenchyma, corresponding to prior instrumentation. Mitochondrial DNA testing was negative. No epilepsy‐specific gene panel, chromosomal microarray, or exome/genome sequencing results were available in the records reviewed for this report. Thus, chronic acquired structural injury related to the prior inflammatory event remains a plausible epileptogenic substrate, but an independent genetic, traumatic, or other epilepsy predisposition cannot be excluded. A simplified clinical and treatment timeline is shown in Figure 3.

Levetiracetam was discontinued because of psychiatric adverse effects, valproic acid because of gastrointestinal intolerance, and topiramate because of inefficacy. He subsequently achieved seizure freedom on cannabidiol (Epidiolex) 540 mg twice daily, clobazam 10 mg twice daily, and lacosamide 200 mg twice daily. At the time of this report, follow‐up after epilepsy onset was approximately 1 year.

## 5 Discussion

Complete or near‐complete clinical and radiologic resolution occurs in more than 70% of pediatric patients with MOGAD‐associated ADEM, and many children recover clinically within 3 months after immunotherapy [6]. Outcomes are nevertheless heterogeneous. Severe attacks, greater lesion burden, and some clinical phenotypes may be associated with persistent neurologic or neurocognitive morbidity [1, 7]. This patient had an exceptionally severe acute course characterized by markedly elevated intracranial pressure, cerebral edema with herniation, extensive MRI abnormalities, prolonged impaired consciousness, and residual cognitive, speech, and learning difficulties.

Acute MOGAD attacks are commonly treated with intravenous corticosteroids, plasma exchange, and/or IVIG, whereas maintenance approaches may include IVIG and other immunotherapies [3, 5]. Early treatment may reduce residual deficits, but optimal acute sequencing, maintenance selection, and treatment duration remain uncertain [5]. In this case, several therapies were used sequentially, so the clinical course cannot establish the effectiveness of any individual agent or demonstrate that maintenance IVIG prevented relapse.

The markedly elevated opening pressure and cerebral edema also required consideration of infectious and CSF‐dynamic mechanisms. Although the infectious evaluation did not identify a pathogen, MOG‐IgG positivity does not by itself exclude a concurrent or preceding infection. Viral infections can trigger post‐infectious inflammatory demyelination, and viral encephalitides, including HSV, VZV, and enteroviral encephalitis, may overlap clinically and radiologically with ADEM‐like presentations. Severe MOGAD‐ADEM itself has also been associated with increased intracranial pressure in pediatric cohorts, particularly in ADEM phenotypes, and may reflect severe inflammatory edema, altered CSF dynamics, or impaired CSF absorption [8]. The choroid plexus is a biologically plausible structure of interest because it forms the blood‐CSF barrier, contributes to CSF production, and participates in immune trafficking and infectious neuroinvasion [9]. In this case, contrast‐enhanced MRI was performed during the acute phase and showed no abnormal enhancement on 4/20/2022; follow‐up contrast‐enhanced MRI on 4/29/2022 showed patchy parenchymal enhancement consistent with subacute lesion evolution but no leptomeningeal enhancement. The available radiology reports did not describe choroid plexus enlargement or enhancement, and the 2025 contrast‐enhanced MRI showed no abnormal intracranial enhancement. Therefore, choroid plexus involvement remains a mechanistic consideration but is not demonstrated in this case.

The later epilepsy requires cautious but anatomically informed interpretation. Rossor et al. reported chronic seizure disorders after ADEM and identified associations with acute symptomatic seizures and other disease features [4]. A conceptual distinction is important: the generalized seizure during the acute encephalopathic MOGAD‐ADEM presentation is best categorized as an acute symptomatic seizure in the setting of active inflammatory disease, whereas the recurrent unprovoked hypermotor seizures 3 years later represent chronic epilepsy in the nonacute phase [10]. This patient had seizures during the acute ADEM episode, bilateral frontal involvement during the acute illness, residual superior frontal abnormalities on earlier follow‐up imaging, and multifocal encephalomalacia/gliosis involving bilateral frontal cortical and subcortical regions on the 2025 post‐epilepsy MRI. These findings make an acquired epileptogenic substrate from prior inflammatory structural injury biologically plausible and electroclinically concordant with the later bifrontal‐predominant epileptiform abnormalities and hypermotor semiology. However, plausibility is not proof of causality. The ictal scalp EEG pattern was diffuse with movement artifact rather than a definitive focal electrographic onset, and the interictal abnormalities were bifrontal‐predominant but multifocal. These limitations preclude definitive attribution of the epilepsy to prior MOGAD‐ADEM or definitive localization as a purely frontal lobe epilepsy.

From a treatment perspective, seizures during active MOGAD are generally managed with immunotherapy directed at the inflammatory attack, together with antiseizure medications when clinically indicated. In contrast, chronic epilepsy after a remote inflammatory event requires renewed assessment for active inflammation, structural injury, and nonimmune etiologies. Current treatment reviews emphasize that immunotherapy may be appropriate when seizures reflect active autoimmune inflammation, whereas postencephalitic or post‐inflammatory structural epilepsy without evidence of active inflammation may not respond to renewed immunotherapy and is typically managed primarily with antiseizure medication strategies [11]. In this patient, the negative MOG‐IgG result at epilepsy onset and lack of documented inflammatory relapse made active MOGAD relapse less evident, although the absence of contemporaneous MRI limits certainty.

An independent genetic epilepsy also remains a meaningful competing explanation. Negative mitochondrial DNA testing does not exclude monogenic, copy‐number, or other genomic causes of epilepsy. Evidence‐based practice guidance recommends considering comprehensive genetic testing in otherwise unexplained epilepsy, with test selection and counseling individualized to the clinical context [12]. In this case, no epilepsy‐focused multigene panel, chromosomal microarray, or exome/genome sequencing result was available. The absence of this information is therefore an etiologic limitation, not evidence against a genetic cause.

The temporal association between the school assault and seizure onset is similarly insufficient to establish isolated post‐traumatic epilepsy. There was no immediate loss of consciousness or altered mentation, and no immediate post‐traumatic imaging was obtained to document or exclude a new traumatic lesion at that time. However, the subsequent 2025 MRI did not show acute infarct, acute intracranial hemorrhage, significant mass effect, hydrocephalus, intracranial mass lesion, or abnormal enhancement; instead, it demonstrated chronic multifocal encephalomalacia/gliosis and diffuse volume loss interpreted as likely sequelae of prior anti‐MOG encephalitis. A transient lowering of seizure threshold in a previously injured brain is therefore conceivable, although not provable. The clinically useful message is that a remote neurologic history should inform, but not replace, a standard evaluation of new‐onset epilepsy. When seizures emerge years after severe inflammatory brain injury, updated neuroimaging, careful electroclinical characterization, review of developmental and family history, and genetic evaluation when clinically appropriate may help distinguish acquired structural, genetic, traumatic, and other etiologies.

Serial MOG‐IgG status should also be interpreted cautiously. Published studies have reported discordant relationships between serostatus and relapse risk [2, 13]. The patient was MOG‐IgG negative when epilepsy emerged, which makes active recurrent MOG‐IgG‐associated inflammation less evident from the available data but does not determine the cause of the epilepsy. MOG‐IgG seronegativity should not be used to infer either that late neurologic symptoms are unrelated to prior injury or that they are a direct complication of MOGAD.

This report is limited by the absence of formal neurocognitive testing results from the tertiary center, lack of oligoclonal band results in the available records, absence of immediate post‐traumatic neuroimaging at the time of later seizure onset, incomplete epilepsy‐focused genetic evaluation, and the inherent inability of a single case to establish treatment effectiveness or causation. The 2025 follow‐up MRI strengthens the plausibility of a chronic acquired structural substrate but was not available as a direct comparison study with the prior outside imaging in the radiology report and does not by itself prove epileptogenic causality. These limitations are central to the interpretation of the case and support a deliberately cautious etiologic conclusion.

## 6 Conclusion

Severe pediatric MOGAD‐associated ADEM can be followed by persistent neurological and neurodevelopmental morbidity despite aggressive treatment. In this patient, epilepsy emerged approximately 3 years later. The subsequent MRI demonstration of multifocal encephalomalacia/gliosis involving bilateral frontal and other cortical–subcortical regions supports chronic acquired structural injury as a plausible epileptogenic substrate, especially given the acute symptomatic seizure during ADEM and later bifrontal‐predominant epileptiform abnormalities. However, definitive causality cannot be established from a single case, and genetic, traumatic, and other epilepsy etiologies remain possible. Longitudinal neurodevelopmental follow‐up after severe MOGAD‐ADEM is important, and remote new‐onset epilepsy should prompt renewed etiologic assessment rather than automatic attribution to the antecedent inflammatory disorder.

## 7 Patient Perspective

The parents described the initial ADEM episode as highly distressing. Following recovery, he experienced persistent neurocognitive, speech, and mild motor deficits that contributed to difficulties with peer relationships and school functioning. He expressed interest in playing sports, although his mother was hesitant to permit participation. At seizure onset, events occurred multiple times daily and required continuous supervision because of safety concerns. With sustained seizure freedom, his mother reported improved confidence in his trajectory and was reconsidering sports participation.

## Author Contributions

**Desiree‐Anne Ramsaran:** conceptualization, writing – original draft, writing – review and editing, data curation. **Garrett Gianneschi:** conceptualization, investigation, writing – original draft, methodology, writing – review and editing, formal analysis, data curation, supervision. **Janet Elgallab:** supervision, writing – original draft, writing – review and editing, conceptualization.

## Funding

The authors have nothing to report.

## Ethics Statement

The authors have nothing to report.

## Consent

Written informed consent for publication was obtained from the patient's parent or legal guardian.

## Conflicts of Interest

The authors declare no conflicts of interest.

## Data Availability

The authors have nothing to report.
